# Undercover Agents of Infection: The Stealth Strategies of T4SS-Equipped Bacterial Pathogens

**DOI:** 10.3390/toxins13100713

**Published:** 2021-10-09

**Authors:** Arthur Bienvenu, Eric Martinez, Matteo Bonazzi

**Affiliations:** Institut de Recherche en Infectiologie de Montpellier (IRIM), Université de Montpellier, CNRS, CEDEX 5, 34293 Montpellier, France; arthur.bienvenu@irim.cnrs.fr

**Keywords:** stealth bacterial pathogens, type 4 secretion system, innate immunity, inflammation, host-pathogen interactions

## Abstract

Intracellular bacterial pathogens establish their replicative niches within membrane-encompassed compartments, called vacuoles. A subset of these bacteria uses a nanochannel called the type 4 secretion system (T4SS) to inject effector proteins that subvert the host cell machinery and drive the biogenesis of these compartments. These bacteria have also developed sophisticated ways of altering the innate immune sensing and response of their host cells, which allow them to cause long-lasting infections and chronic diseases. This review covers the mechanisms employed by intravacuolar pathogens to escape innate immune sensing and how Type 4-secreted bacterial effectors manipulate host cell mechanisms to allow the persistence of bacteria.

## 1. Introduction

Gram-negative bacteria use complex nanomachines, called secretion systems, to translocate effector proteins, molecules and DNA in order to modulate the microbial response to the environment. Nine secretion systems have been identified to date: five span the inner and outer bacterial membrane, while the remaining four are embedded in the outer membrane [1]. Initially described in the context of bacterial conjugation and horizontal gene transfer, type 4 secretion systems (T4SS) are large protein complexes that span through the inner and outer membranes of several Gram-negative and Gram-positive bacteria [2]. These highly versatile nanomachines are characterised by an internal channel that mediates the translocation of proteins, toxins and genetic material. Cytoplasmic ATPases typically energise translocation. Depending on their function, T4SS can be divided into three functional groups [3]. T4SSs belonging to the first group mediate the conjugative transfer of DNA and transposon among bacteria, or from bacteria to plants, fungi and mammalian cells. Notably, DNA exchange has been associated with genome plasticity, the acquisition of eukaryotic domains and the acquisition of antibiotic resistance traits [3]. T4SSs belonging to the second group mediate the release and uptake of DNA directly from the extracellular milieu. Finally, the third group of T4SS mediates the translocation of proteins. These are primarily found in pathogenic bacteria and are essential virulence factors that coordinate host/pathogen interactions. Interestingly, T4SSs are not exclusive, and some bacteria, including *Helicobacter pylori*, encode both a DNA exchange system and a protein delivery system [4]. Genes encoding T4SSs are usually arranged in operons or clusters of operons [2]. The type 4a secretion system (T4SSa, also known as VirB/D4 secretion system) was initially discovered in the plant pathogen *Agrobacterium tumefaciens* and is usually composed of 12 genes [5]. Bacterial pathogens, including *Orientia tsutsugamushi*, *Brucella* spp., *Bartonella* spp., *Anaplasma* spp. and *Ehrlichia* spp., possess a T4SSa [6]. *Bartonella* species, except *B. bacilliformis*, can also express additional T4SSs, such as the Vbh/TraG and Trw secretion systems, that play a role in bacterial conjugation and erythrocyte invasion, respectively [7]. The T4SSb (also known as the Dot/Icm secretion system) is composed of 23 to 25 genes, and it is only found in *Legionella* and *Coxiella* species [8].

Notably, many bacterial pathogens equipped with a type 4 secretion system (T4SS) have evolved to colonise host cells from the safe haven of replicative vacuoles. These membrane-bound compartments derive from the initial internalisation vacuole that hosts the pathogen and are de facto intracellular compartments of eukaryotic composition, which only exist in the context of infection. Life in a membrane-enclosed compartment presents several advantages, including access to nutrients and escape from cytosolic immune surveillance. On the other hand, without bacterial intervention, internalisation vacuoles fuse with lysosomes, which in most cases is fatal for the pathogen itself. Thus, these pathogens rely on their T4SS to translocate effector proteins through the membrane of the replicative vacuole, directly into the host cytoplasm, where they subvert membrane trafficking to deviate their replicative niche off the tracks of the endocytic pathway [9]. Each replicative niche is remarkably specific to its pathogen and is characterised by a unique protein and lipid signature and morphology. Furthermore, the “fate” of these compartments may differ depending on the pathogen’s lifestyle: some remain intact and host non-replicative bacteria for an extended time, whereas others rupture to release bacteria and facilitate the infection of bystander cells. Importantly, infected cells are not entirely blind to these pathogens, which are readily recognised by cell surface and endosomal Toll-like receptors (TLRs) that transduce signals leading to the activation of the antimicrobial response [10]. Later, during the intracellular cycle, cytosolic receptors sense pathogen-derived ligands, which may include the effector proteins themselves [11]. Furthermore, infected cells can destabilise the membrane of the bacterial replicative niche, thereby exposing microbes to immune surveillance [12]. Finally, innate immunity can also be alerted by recognition of the hybrid membranes of replicative vacuoles as foreign bodies [13]. Thus, several T4SS pathogens have evolved stealth strategies to remain undetected within infected cells and avoid triggering inflammation.

This review illustrates the diverse strategies developed by stealth pathogens equipped with a T4SS to elude innate immune recognition and/or silence the multiple signalling pathways coordinating the inflammatory response.

## 2. Escape from Host Sensing

**Figure 1:**
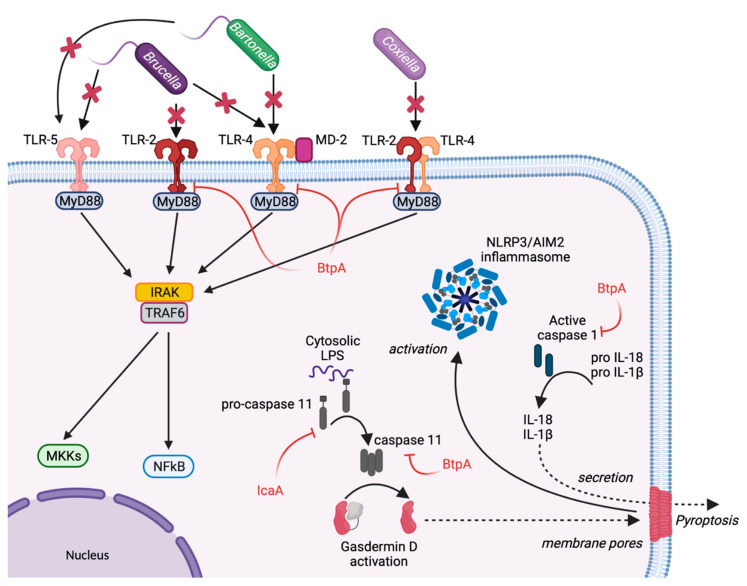
Pathogen evasion of host cell sensing. Pathogen recognition is essential to initiate an inflammatory response. Due to their specific LPS structure, *Brucella* spp., *Bartonella* spp. and *C. burnetii* are poorly recognised by TLRs. Furthermore, *Brucella melitensis* and *Bartonella bacilliformis* flagellin are weak activators of TLR5. By competing with the interaction of TLRs with MyD88, the *Brucella* effector protein BtpA is the only bacterial protein currently known to directly inhibit the TLR signalling pathway. BtpA also induces caspases 1, 4 and 11 degradation, impairing interleukin secretion. The *C. burnetii* effector protein IcaA actively modulates the inflammatory response by inhibiting the activation of caspase 11.

In immune cells, the surveillance and detection of invading bacterial pathogens are ensured by pattern-recognition receptors (PRR), such as surface and endosomal TLRs and cytosolic Nucleotide oligomerization domain (NOD)-like receptors (NLRs) [14]. The NLR family of proteins comprises 22 members, who are critical in triggering and modulating the inflammatory response of immune cells. TLRs are transmembrane proteins that localise either at the cell surface or at the membranes of intracellular compartments, such as the endoplasmic reticulum (ER), endosomes, lysosomes, or endolysosomes. The human genome encodes 10 proteins of the TLR family. They recognise distinct or overlapping pathogens associated molecular patterns (PAMPs), such as lipids, lipoproteins, proteins, and nucleic acid [15]. Each TLR comprises an ectodomain with leucine-rich repeats (LRRs) that mediate PAMPs recognition, a transmembrane domain, and a cytoplasmic Toll/Interleukin-1 (IL-1) receptor (TIR) domain that initiates downstream signalling. Some stealth bacteria adapted their microbe-associated molecular patterns (MAMPs) to escape recognition by cell sensors. For example, the *Bartonella* lipid A is poorly recognised by TLR4 as its structure is characterised by a short glycosyl backbone (di-aminoglucose), a pentaacylation, and a very long fatty acid side chain [16]. This lipid A impedes a broad inflammatory response [17]. Furthermore, *B. quintana* Lipopolysaccharide (LPS) is a potent antagonist of TLR4 signalling (Figure 1) [18,19].

*Brucella* LPS exhibits low toxicity, and its atypical structure was postulated to delay the host immune response, favouring the establishment of chronic disease [20]. *Brucella* Lipid A is a 2,3-diaminoglucose disaccharide substituted with C16, C18, C28 and other very long acyl chains [21]. This peculiar structure is a poor agonist of TLR4/myeloid differentiation-2 (MD-2), and a paradigm has emerged proposing *Brucella* LPS as a crucial virulence factor that hampers recognition by PRRs and plays essential roles during infection (Figure 1) [16]. One study showed that *Brucella* LPS does not induce inflammatory responses in macrophages and dendritic cells (DCs), two of the most important immune system sentinels. This was attributed to its poor recognition by TLR4/MD-2, which is widely considered the major receptor complex for LPS binding and signalling [22].

As previously mentioned, the activation of TLR4 and TLR2 is dependent on strong affinity and direct interaction with bacterial LPS. In macrophages challenged with avirulent *Coxiella burnetii* LPS, TLR-4 and TLR-2 co-immunoprecipitate, indicating that the avirulent strain can be recognised by these receptors. However, this association was absent in cells challenged by the LPS of virulent *C. burnetii* (Figure 1). The disruption makes TLRs unable to signal during the recognition of the LPS of pathogenic *C. burnetii* [23]. The re-organization of the macrophage cytoskeleton induced the disruption of TLR-2 and TLR-4 by *C. burnetii* LPS. Interestingly, blocking the actin cytoskeleton re-organization relieved the disruption of the association TLR-2/TLR-4 by pathogenic *C. burnetii* and rescued the p38α-mitogen-activated protein kinase (MAPK) activation by *C. burnetii* [23].

Bacterial flagellin is another major MAMP that can be sensed by TLR5 and NLR family CARD domain containing 4 (NLRC4), leading to a robust pro-inflammatory response. While flagellin from *Brucella melitensis* is weakly detected by TLR5 and might play an essential role in immune escape [24], *Bartonella bacilliformis* synthesises flagellin molecules that escape TLR5 recognition entirely (Figure 1). This is due to the presence of aspartic acid and serine amino acids in the 89–96 region of *N*-terminal D1 domain of flagellin, which is usually recognised by TLR5. These changes in amino acids prevent TLR sensing while preserving bacterial motility [25].

Besides eluding TLR recognition, *Brucella* uses additional evasion strategies by suppressing innate immune signalling. The *B. abortus* Btp1 effector protein and its *B. melitensis* homolog TcpB (now both referred to as BtpA) encode a TIR domain that competes with myeloid differentiation response gene 88 (MyD88) for binding with TIR domain-containing adaptor protein (TIRAP, Figure 1), which ultimately facilitates the ubiquitination and degradation of MyD88 adaptor-like (Mal) and inhibits both TLR4 and TLR2 signalling [26,27]. TcpB seems to be important in the bacterial immune escape, as the *B. melitensis tcpB* mutant displays a markedly retarded systemic spread in a mouse model of infection. The weakened phenotype of *tcpB* mutants for immune-competent mice suggests that *Brucella* can evade innate immunity by using multiple strategies [28]. Finally, the *Brucella* effector BtpB inhibits TLR signalling and contributes to the control of dendritic cell activation [29]. Recently, BtpA and BtpB have been shown to modulate energy metabolism through NAD+ hydrolysis, which could in part explain the down-modulation of innate immune signalling in *Brucella*-infected cells [30].

## 3. Inhibition of Inflammasome

Upon detection of PAMPs, several NLRs (such as NLR family Pyrin Domain containing 1 (NLRP1), NLR family Pyrin Domain containing 3 (NRLP3) or Pyrin) oligomerise with the apoptosis-associated speck-like protein containing a CARD (ASC) to activate inflammatory Caspase-1 [11]. The major Caspase-1 substrates are Gasdermin-D (GSDMD), pro-IL-1β, and pro-IL-18. Cleaved GSDMD targets and permeabilizes the plasma membrane, thus triggering ion imbalance and necrotic cell death, which is termed “pyroptosis” [31]. During this process, cleaved IL-1β and IL-18 are also secreted and stimulate inflammatory responses in neighbouring cells. Cytosolic LPS can be sensed directly by Caspase-11 (in mouse cells) and Caspase-4 and -5 (in human cells), which leads to GSDMD cleavage and pyroptosis [32]. These caspases belong to the non-canonical inflammasome pathway as they cannot directly process the inflammatory cytokines IL-1β and IL-18 [33].

*Brucella* effector BtpA interacts with Caspase-4 and induces the ubiquitination of inflammatory caspases-1, 4, and 11, leading to their degradation [34]. BtpA expression leads to a reduction of IL-1β secretion, induced by the non-canonical inflammasome in *Brucella*-infected macrophages (Figure 1). BtpA also attenuates pyroptosis and proinflammatory cytokine secretion in macrophages infected with *Salmonella enterica* [34].

*C. burnetii* does not stimulate inflammasome activation in primary mouse macrophages, as Caspase-1 is not activated during infection [35]. The secretion of the effector protein IcaA inhibits the non-canonical caspase-11-mediated activation of the NLRP3 inflammasome (Figure 1). However, the molecular targets of this effector protein remain unknown [35]. Other *C. burnetii* mechanisms may be involved in the inhibition of inflammation as several *Coxiella* transposon mutants displayed a cytotoxic phenotype during infection [36].

## 4. Inhibition of Transcription and Translation

The recognition of danger-associated molecular patterns (DAMPs) by infected cells triggers an important transcriptional reprogramming, which is essential to mount a specific proinflammatory response. The three primary inflammation signal-transducing pathways are the signal transducers and activators of transcription (STATs), the interferon regulatory factors (IRFs), and the nuclear factor κB (NFκB) [37]. To counter this, stealth pathogens effectively subvert the host transcriptional response, either by epigenetic silencing or by perturbing the nuclear accumulation of the main transcriptional regulators.

**Figure 2:**
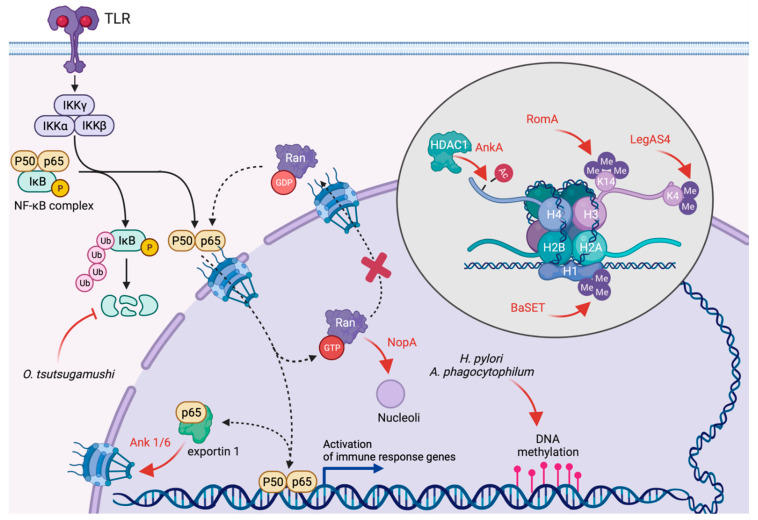
Modulation of the host transcriptional landscape by bacterial pathogens. T4SS-equipped bacterial pathogens manipulate the host transcriptional response to infection by subverting the NF-kB signalling pathway or remodelling the host epigenome. Unidentified *O. tsutsugamushi* effector proteins stabilise IkB, thereby preventing NF-kB translocation to the nucleus. Furthermore, this microbe also translocates the ankyrin repeat effector proteins Ank1 and Ank6, promoting the nuclear export of p65. On the contrary, the *C. burnetii* effector protein NopA sequesters Ran at nucleoli, thereby perturbing nucleocytoplasmic traffic preventing the nuclear import of p65 and IRF3 transcription factors. Infections by *H. pylori* and *A. phagocytophilum* trigger an increase in DNA methylation, silencing transcription, whereas other bacteria specifically target histones after translational modifications. The *L. pneumophila* effector RomA/LegAS4 targets histone H3 methylation. RomA promotes trimethylation at lysine 14, whereas LegAS4 promotes the dimethylation of lysine 4. The *B. anthracis* effector BaSET promotes trimethylation of histone H1, whereas the *A. phagocytophilum* effector AnkA subverts HDAC1 function, promoting histone de-acetylation.

Controlling DNA methylation and histones after -translational modifications are efficient strategies to control the transcriptional landscape of infected cells. Thus, over the past decade, epigenetic silencing of host defences has emerged as a common strategy of several bacterial pathogens. DNA methylation interferes with the recruitment of transcription factors and leads to transcriptional repression [38]. Global changes in the methylation state of intergenic DNA regions have been described in cells infected by *Helicobacter pylori* [39] or *Anaplasma phagocytophilum* [40] (Figure 2). In the latter case, this is required for optimal intracellular replication. However, whether DNA methylation is actively triggered by bacterial effectors or reflects the cellular response to infection remains to be defined.

Histone modifications include acetylation, methylation and phosphorylation. Collectively, they regulate the accessibility of DNA to transcription factors, thus regulating gene expression [41]. Enzymatically active bacterial effectors can modulate epigenetic histone marks directly. Examples include an emerging class of bacterial effectors encoding a eukaryotic-like suppression of variegation, enhancer of zeste and trithorax (SET) domain with methyltransferase activity. *Legionella pneumophila* translocates the effector protein RomA/LegAS4 [42,43], which localises to the nucleus of cells infected with the Paris strain, an endemic *Legionella* strain predominant in France [44], but displays a marked nucleolar localisation when cells are infected with *L. pneumophila* Lp02, a virulent thymine auxotroph derived from the serogroup 1 Philadelphia-1 strain [45]. Interestingly, despite their high homology, RomA catalyses the tri-methylation of histone H3 at lysine 14 (Figure 2), which results in the repression of host innate immune genes, such as Tumor Necrosis Factor (TNF), IL-6, TLR5 and NLRP3 [42]. On the contrary, LegAS4 catalyses a di-methylation of histone H3 at lysine 4, leading to increased transcription of rDNA genes [43] (Figure 2). Similar to RomA, the *Bacillus anthracis* protein BaSET encodes a SET domain and localises to the nucleus of infected cells and specifically tri-methylates histone H1 [46] (Figure 2). The ectopic expression of BaSET represses the activity of NF-kB response elements, including the pro-inflammatory cytokines IL-6 and IL-8 [46]. Furthermore, BaSET is important for virulence as assessed using a murine bacteremia model [46]. However, whether BaSET is a T4SS substrate remains to be defined. Of note, SET domain proteins have been identified in other bacterial pathogens, including *Chlamydia* spp. [47] and *Burkholderia thailandensis* [43], which encode type 3 and type 6 secretion systems, respectively.

Other bacterial effector proteins indirectly manipulate histone marks. Among T4SS-equipped pathogens, the best example is the *A. phagocytophilum* effector protein AnkA, which translocates into the nucleus of infected cells and directly interacts with the histone deacetylase HDAC1 [48] (Figure 2). The removal of acetyl groups from histones mediated by HDACs favours a closed conformation of chromatin, thereby terminating transcription. Importantly, AnkA also binds host DNA at ATC-rich intergenic regions upstream of genes involved in the antimicrobial immune response [49], targeting HDAC1 activity towards the specific silencing of host defence genes [50]. Interestingly, *A. phagocytophilum* infections also increase the expression of HDAC through an unknown mechanism [50].

The mammalian NF-κB complex consists of five proteins that function by forming homo- or heterodimers. Among these, the transcription factor p65 is retained in the cytosol by the interaction with members of the NF-κB inhibitor (IκB) family, which hides its nuclear localisation signal (NLS). Exposure to PAMPs or TNFα activates a signalling cascade that leads to the phosphorylation and proteasomal degradation of IκB proteins. This exposes the NLS on p65, which translocates into the nucleus and coordinates the expression of antimicrobial and pro-inflammatory genes, such as Interleukin-1α (IL-1α), Interleukin-1β (IL-1β), Interleukin-6 (IL-6), etc (Figure 2). Several bacterial pathogens interfere directly with this signalling cascade at different levels, thereby activating or inhibiting the NF-κB pathway [51].

Emerging evidence indicates that T4SS-equipped microbes translocate effector proteins that manipulate the NF-κB pathway indirectly, by subverting the host nucleocytoplasmic transport. The *Orientia tsutsugamushi* ankyrin repeat-containing effectors Ank1 and Ank6 are transported to the nucleus of infected cells in an importin β1-dependent manner [52]. There, they interact with and rely on the function of exportin 1 to promote p65 nuclear export [52] (Figure 2). The cytoplasmic accumulation of p65 should be sufficient to reduce NF-κB-dependent transcriptional activation. Interestingly, however, preventing nuclear export in *O. tsutsugamushi*-infected cells does not restore NF-κB-dependent transcription [52], suggesting the existence of redundant strategies to dampen the innate immune response to *Orientia* infections. Indeed, it has recently been reported that *O. tsutsugamushi* also stabilises the intracellular levels of the IκB family member p105, thereby fostering p65 cytoplasmic retention (Figure 2). While the molecular mechanisms regulating this phenomenon remain to be defined, p105 stabilisation depends on the bacterial load and protein synthesis, suggesting the implication of effector proteins [53]. Similar to *Orientia*, *C. burnetii* also perturbs nucleocytoplasmic traffic to dampen the NF-κB-dependent transcriptional response to infection. The effector protein NopA (for nucleolar protein A) localises at the nucleoli of infected cells, interacts with the eukaryotic small GTPase Ran, and sequesters it within the nucleus [54] (Figure 2). As nucleocytoplasmic transport depends on a protein concentration gradient of Ran between the cytoplasm and the nucleus [55], NopA activity effectively perturbs the nuclear import of proteins, including the transcription factors p65 and interferon regulatory transcription factor 3 (IRF3) (Figure 2). Consequently, cytokines expression is strongly attenuated in cells infected by wild-type *Coxiella*, whereas this effect is completely lost in cells infected either with *nopA* or T4SS-defective *dotA* mutants [54]. Notably, despite their subcellular localisation, neither Ank1, Ank6, nor NopA encode canonical nuclear localisation signals (NLS), suggesting that bacterial effector proteins have evolved alternative strategies to target subcellular compartments.

Parallel to the NF-κB pathway, and besides its role in cell development and differentiation, the Janus Kinase (JAK)-Signal Transducer and Activator of Transcription (STAT) signalling pathway is central to the host cell’s response to viral and bacterial infections. Signal transducer and activator of transcription 3 (STAT3) is a key regulator of the switch between pro- and anti-inflammatory signalling in cells [56]. In its inactive form, STAT3 is found as a monomer in the cytoplasm. Cellular stressors, including cytokines, activate surface receptors and transduce the signal to cytoplasmic Janus kinases (JAKs), which phosphorylate STAT3, triggering its dimerisation and nuclear translocation. Notably, STAT3 can also be phosphorylated by non-receptor tyrosine kinases, including MAP kinases, Src and c-Abl. The *Bartonella henselae* effector protein BepD possesses several EPIYA-related motifs that can be phosphorylated by host Src-family tyrosine kinases. Importantly, this leads to the recruitment of STAT3 and facilitates its activation by c-Abl-dependent phosphorylation, thereby bypassing the canonical JAK pathway. BepD-mediated activation of the STAT3 pathway leads to impairment of pro-inflammatory TNFα secretion and stimulation of the anti-inflammatory cytokine Interleukine-10 (IL-10) [57].

## 5. Modulation of the Unfolded Protein Response

The unfolded protein response (UPR) also plays a relevant role in the interplay between pathogens and inflammation. UPR is triggered by stress conditions at the level of the ER in response to the accumulation of unfolded or misfolded proteins in the ER lumen due to an increase of protein secretion or a direct disruption of ER protein folding [58]. UPR includes multiple signal transduction pathways that regulate gene transcription, protein modifications, and mRNA translation to restore cellular homeostasis. Rapid modifications of the transcriptional profile of cells exposed to pathogens can trigger UPR. Three main pathways have been identified and depend on three ER transmembrane protein sensors: inositol-requiring enzyme-1α (IRE1α), protein kinase RNA-like endoplasmic reticulum kinase (PERK), and activating transcription factor 6α (ATF6α) [59]. The failure of the UPR to restore cellular homeostasis results in apoptosis. Notably, some intravacuolar pathogens, including *Brucella* spp. and *L. pneumophila*, escape the endocytic maturation pathway by favouring interactions between the membranes of their replicative niches with those of the ER, which can be sensed as an ER stress. UPR induction may be triggered explicitly by effector proteins and may be required for intracellular replication, as shown for the *Brucella* effector proteins VceC and TcpB [60,61]. During *L. pneumophila* infections, the IRE1α pathway is activated by TLRs upon extracellular pathogen sensing. However, *L. pneumophila* silences the downstream UPR pathways by two different mechanisms involving the effector proteins Lgt1 and Lgt2, which inhibit the splicing of XBP1u to XBP1s, thereby blocking the IRE1 pathway and unidentified effector proteins that inhibit the translation of ATF6-upregulated genes, leading to an inhibition of the cell response [62,63]. Interestingly, infections by *C. burnetii* also activate the UPR, as indicated by an increase in the intracellular levels of CCAAT/enhancer binding proteins (C/EBPs) homologous protein (CHOP). As for *Brucella* infections, UPR activation is required for optimal *Coxiella*-containing vacuole (CCV) expansion. However, unidentified *C. burnetii* effectors actively prevent CHOP nuclear translocation and downstream apoptosis [64]. Furthermore, the intracellular localisation of the *C. burnetii* effector CaeB has been recently re-assessed, and it has been shown that the effector localises at the ER upon ectopic expression. CaeB specifically modulates IRE1 signalling in cells exposed to tunicamycin, which ultimately leads to the inhibition of apoptosis [65]. This is in line with previous observations on the anti-apoptotic properties of CaeB in cells exposed to staurosporine, which is mediated by an inhibition of mitochondrial outer membrane permeabilisation (MOMP) [66]. Finally, another aspect of the UPR pathway is the stress response leading to the ER-associated degradation (ERAD) of misfolded proteins. Ank4, an effector protein of *O. tsutsugamushi*, has been shown to interact with HLA-B-associated transcript 3 (Bat3) to impair ERAD mechanism during the early stages of infection. Bat3 is an essential protein of ERAD that acts as a chaperone to mediate the translocation of misfolded proteins to the cytosol, where they can be ubiquitinated prior to degradation by the proteasome [67]. *O. tsutsugamushi* temporarily blocks ERAD until ERAD-derived amino acids are needed to support its growth [68].

## 6. Subversion of Autophagy

**Figure 3:**
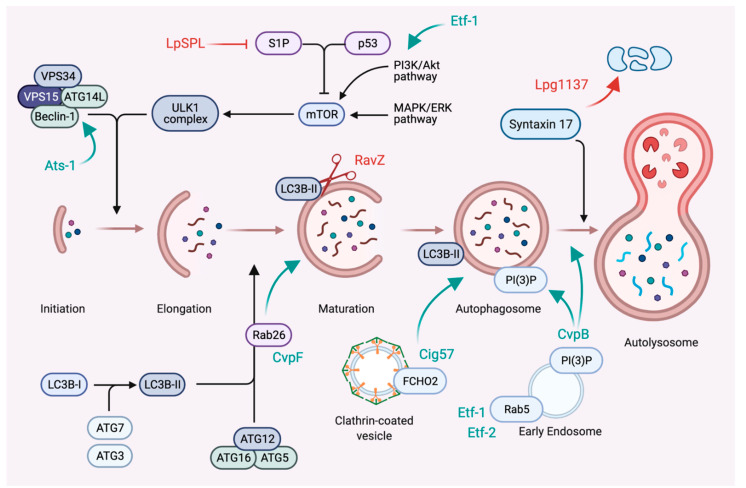
Subversion of autophagy by stealth bacterial pathogens. Stealth pathogens equipped with a T4SS translocate effector proteins to either activate or inhibit autophagy and establish infections. *L. pneumophila* inhibits autophagy, thus allowing LCVs to escape the degradative pathway. LpSPL downregulates host sphingolipid levels to perturb autophagy initiation; RavZ irreversibly cleaves the autophagy adapter protein LC3B from autophagosomes to inhibit autophagosomes maturation, and Lpg1137 degrades Syntaxin 17 to inhibit the fusion of autophagosomes with lysosomes. *C. burnetii* promotes autophagy, probably to reroute membranes and nutrients to CCVs. The effector protein CvpF enhances the Rab26-mediated recruitment of LC3B to autophagosomes; Cig57 stimulates the clathrin-dependent recruitment of autophagy markers to CCVs and CvpB manipulates PI(3)P metabolism to enhance autophagosomes fusion with CCVs, thereby favouring their homotypic fusion. *E. chaffeensis* also stimulates autophagy by secreting the effectors Etf-1 and Etf-2, which activate class III phosphatidylinositol 3-kinase and Rab5. Finally, the *A. phagocytophilum* effector protein Ats-1 interacts with Beclin-1 and ATG14L to stimulate autophagosome formation.

Macroautophagy (hereafter referred to as autophagy) is a eukaryotic process for the engulfment and degradation of cytosolic material, including misfolded proteins, damaged organelles and macromolecules [69]. While basal autophagy is crucial to maintain cellular homeostasis by providing energy substrates to the cell, it plays an important role in the innate immune response against intracellular pathogens. Intravacuolar pathogens developed strategies to either avoid degradation by the autophagic machinery or divert autophagy elements to generate their replicative niche. *L. pneumophila* uses effector proteins to inhibit autophagy and avoid destruction in lytic autolysosomes. The bacterium secretes the cysteine protease RavZ that specifically and irreversibly cleaves the autophagy adapter protein LC3B from autophagosomes, allowing the *Legionella*-containing vacuoles (LCVs) to escape degradation by the autophagy machinery (Figure 3) [70]. Interestingly, LCVs generated by Δ*ravZ* mutants still lack the autophagy marker LC3B, strongly suggesting that the bacterium secretes additional effectors capable of diverting the autophagy machinery away from the *Legionella* replicative niche. *L. pneumophila* also secretes Lpg1137, a serine protease that triggers the degradation of Syntaxin 17 (Stx17), a SNARE involved in vesicle trafficking and autophagy [71]. Additionally, some *Legionella* strains exert additional control over the autophagy machinery via host sphingosine-1-phosphate lipid levels (Figure 3). This lipid mediates the balance between sphingolipid-induced autophagy and cell death. *L. pneumophila* secretes the effector LpSPL (Sphingosine-1 phosphate lyase) to downregulate host sphingolipid levels, which causes a delay in the autophagic response to *L. pneumophila* infections [72]. *A. phagocytophilum* and *Ehrlichia chaffeensis* hijack the autophagosome initiation machinery to develop inside vacuoles with autophagosomal properties. *A. phagocytophilum* secretes the effector protein Ats-1, which interacts with Beclin-1 and ATG14L to induce autophagosome formation [73]. *E. chaffeensis* secrete Etf-1, which stimulates autophagy by activating class III phosphatidylinositol 3-kinase and Rab5 [74], and Etf-2, which binds to active Rab5 and delays endosomal maturation (Figure 3) [75]. For these two intracellular bacteria, the diversion of autophagy seems devoted to the acquisition of nutrients and membranes, as their respective vacuoles do not fuse with lysosomes and avoid being acidified. *C. burnetii* appears unique in the group of intravacuolar pathogens as the biogenesis of its vacuole relies on the autophagy machinery and fusion with lysosomes [76,77]. *C. burnetii* secretes several effector proteins to manipulate autophagy and promote CCV biogenesis and expansion. *Coxiella* vacuolar protein B (CvpB) localises to CCVs, where it stabilises the host lipid phosphatidylinositol 3-phosphate (PI(3)P) to stimulate autophagy and the homotypic fusion of CCVs [78,79,80]. Recently, effector protein CvpF was shown to stimulate Rab26-dependent autophagy to expand *Coxiella*-containing vacuoles [81]. Finally, the effector protein Cig57 interacts with the clathrin accessory protein FCHO2 [82] and stimulates the clathrin-dependent recruitment of autophagy markers to CCVs (Figure 3) [83]. In the case of *Brucella*, the autophagy initiation machinery intervenes at the end of the bacterial cell cycle to facilitate egress of the bacterium [84]. Interestingly, the autophagy adapter LC3B is upregulated during *Brucella* infections of human peripheral blood mononuclear cells (PBMCs). *Brucella*-induced autophagy leads to a defect in monocyte polarisation, as these cells fail to differentiate into M1 (pro-inflammatory) or M2 (anti-inflammatory) macrophages [85], suggesting that autophagy upregulation could favour the bacterial escape from a controlled immune response.

## 7. Inhibition of Apoptosis

**Figure 4:**
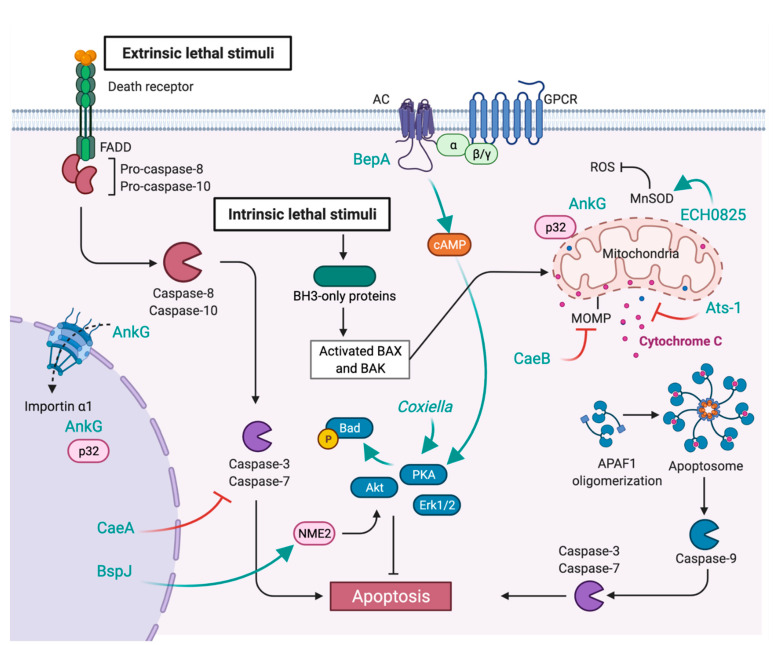
Subversion of apoptosis by stealth bacterial pathogens. Stealth pathogens inhibit apoptosis of infected cells to establish persistent infections. Unidentified *C. burnetii* effectors stimulate the activity of the anti-apoptotic factors PKA, Akt and Erk1/2. The effector protein AnkG localises at mitochondria but can translocate to the nucleus where it interacts with the proapoptotic factor p32. The effector proteins CaeA and B inhibit extrinsic and intrinsic apoptosis by preventing the activation of caspase 7 and by inhibiting mitochondrial outer membrane permeabilisation, respectively. The *Brucella* effector BspJ could interact with NME2 and CKB (not shown) to inhibit macrophage apoptosis. The *Ehrlichia* effector ECH0825 localises to the mitochondria, where it upregulates MnSOD, thereby decreasing ROS-mediated apoptosis. The *Anaplasma* effector protein Ats-1 translocates to mitochondria, where it inhibits cytochrome c release. Finally, the *Bartonella* BepA effector binds the adenylyl cyclase (AC), thereby stabilising its interaction with the G-protein-coupled receptor (GPCR) subunit, Gαs. This leads to an increase in cellular cAMP levels, protecting endothelial cells from apoptosis.

Programmed cell death is common to all multicellular organisms. It is essential to dispose of unwanted cells, either during development or disease. Apoptosis can be activated by the extrinsic pathway upon sensing environmental stress by cell surface-exposed death receptors or by the intrinsic pathway, which detects intracellular stress (DNA damage, starvation, hypoxia, etc.) (Figure 4) [86]. Both pathways lead to the activation of caspases to coordinate intracellular proteolytic events. The intrinsic pathway also promotes mitochondrial outer membrane permeabilisation (MOMP), followed by cytochrome C release, which initiates the apoptosome assembly. Several intravacuolar pathogens promote the lysis of infected cells to propagate infections; however, host cell death by apoptosis is important to control intracellular bacteria replication and survival. Thus, stealth pathogens translocate effector proteins to prevent untimely cell death and preserve their replicative niche during chronic infections. The inhibition of apoptosis by *C. burnetii* is particularly well detailed and stands as a remarkable example of how important host/pathogen interaction hubs can be multilayered and regulated by redundant mechanisms. Indeed, *C. burnetii* infections trigger an anti-apoptotic transcriptional program [87] and the activation of pro-survival kinases, including Akt, Erk1/2, and cAMP-dependent protein kinase (PKA) [88,89]. Activated PKA phosphorylates the pro-apoptotic protein Bad, which is sequestered at CCVs during infections. While this process relies on a functional T4SS, the effector protein/s involved remain unidentified [88]. *C. burnetii* possesses at least 3 effector proteins capable of inhibiting apoptosis. AnkG inhibits apoptosis by interacting with the proapoptotic mammalian protein p32 (Figure 4) [90]. Ectopically expressed AnkG associates with mitochondria but traffics to the nucleus after staurosporine-induced apoptosis. AnkG lacks typical NLS; however, binding to p32 and importin-α1 is required for nuclear translocation, suggesting that the effector protein might piggyback on host proteins [91,92]. How AnkG prevents apoptosis remains to be defined. Besides AnkG, *Coxiella* anti-apoptotic effectors A and B (CaeA and CaeB, respectively) block both intrinsic and extrinsic apoptosis (Figure 4). As already discussed above, CaeB localises at the ER, targets the ER stress sensors IRE1 [65] and prevents MOMP [66]. Ectopically expressed CaeA localises at the nucleus and prevents the activation of executioner caspase 7 [93]. Although the molecular mechanisms regulating CaeA function remain to be described, its antiapoptotic effect depends on the number of EK repetitions embedded in its protein sequence, which is variable among *Coxiella* isolates [93]. While the secretion of this protein still needs to be confirmed, the *Brucella abortus* protein BspJ also localises at nuclei upon ectopic expression and is important for the intracellular survival of the bacterium. Preliminary experiments indicate that BspJ could interact with NME/NM23 nucleoside diphosphate kinase 2 (NME2) and creatine kinase B (CKB) to inhibit macrophage apoptosis [94]. *Ehrlichia* and *Anaplasma* target mitochondria to prevent apoptosis during infections. The *Ehrlichia* type IV effector ECH0825 is highly upregulated during exponential growth in human monocytes and localises to the mitochondria upon T4SS-mediated translocation (Figure 4). ECH0825 upregulates mitochondrial manganese superoxide dismutase (MnSOD) to decrease reactive oxygen species (ROS) and dampen ROS-mediated cellular damages and apoptosis [95]. The *Anaplasma* effector protein Ats-1 has a dual role in apoptosis and autophagy. The protein translocates to the mitochondria, where it is cleaved by host proteases and inhibits cytochrome c release and poly ADP-ribose polymerase (PARP) cleavage [96]. Finally, the *Bartonella* BepA effector binds the C2 catalytic domain of host adenylyl cyclase (AC) and stabilises its interaction with G protein-coupled receptor (GPCR) subunit Gαs. The latter stimulates the activity of AC, leading to an increase in cellular cAMP levels, which protect endothelial cells from apoptosis [97,98].

## 8. Concluding Remarks

Infections by pathogens are typically characterised by two possible outcomes: pathogens are either cleared by an efficient innate immune response, pre-existing immunity and, of course, medical treatment; or pathogens overcome host defences, eventually killing their hosts. Either way, infections typically manifest with detectable symptoms that allow a rapid response. However, a number of pathogens, including viruses, fungi and bacteria, have adapted to their hosts in such a way that they evade innate immune recognition and/or suppress the downstream signalling pathways that lead to the mounting of an antimicrobial response. These microorganisms are thus called stealth pathogens and can be the cause of long-lasting chronic infections that often remain undiagnosed. Notably, many stealth bacteria are equipped with a T4SS and replicate within membrane-bound compartments whose biogenesis relies on the subversion of host cell membrane trafficking by bacterial effector proteins. Interestingly, however, despite their shared lifestyle and secretion system, stealth bacteria target an extremely diverse variety of host pathways involved in mounting an inflammatory response. In some cases, multiple effector proteins target the same pathway, suggesting the existence of fail-safe mechanisms. Understanding how stealth pathogens manipulate the host immune response is of utmost importance to counter latent chronic infections, both by developing specialised diagnostic tools to improve detection, and by specifically boosting adaptive immunity to clear these pathogens. On the other hand, understanding the mode of action of stealth pathogen effector proteins may provide us with innovative tools to modulate the immune system in the context of non-infectious chronic diseases.

## Data Availability

Not applicable.
